# Parvalbumin-positive neurons in the medial vestibular nucleus contribute to vestibular compensation through commissural inhibition

**DOI:** 10.3389/fncel.2023.1260243

**Published:** 2023-11-08

**Authors:** Yuejin Zhang, Guangpin Chu, Yangming Leng, Xueling Lin, Hong Zhou, Yisheng Lu, Bo Liu

**Affiliations:** ^1^Department of Otorhinolaryngology, Union Hospital, Tongji Medical College, Huazhong University of Science and Technology, Wuhan, China; ^2^Department of Physiology, School of Basic Medicine, Tongji Medical College, Huazhong University of Science and Technology, Wuhan, China

**Keywords:** vestibular compensation, unilateral labyrinthectomy, commissural inhibitory system, medial vestibular nucleus, parvalbumin

## Abstract

**Background:**

The commissural inhibitory system between the bilateral medial vestibular nucleus (MVN) plays a key role in vestibular compensation. Calcium-binding protein parvalbumin (PV) is expressed in MVN GABAergic neurons. Whether these neurons are involved in vestibular compensation is still unknown.

**Methods:**

After unilateral labyrinthectomy (UL), we measured the activity of MVN PV neurons by *in vivo* calcium imaging, and observed the projection of MVN PV neurons by retrograde neural tracing. After regulating PV neurons’ activity by chemogenetic technique, the effects on vestibular compensation were evaluated by behavior analysis.

**Results:**

We found PV expression and the activity of PV neurons in contralateral but not ipsilateral MVN increased 6 h following UL. ErbB4 is required to maintain GABA release for PV neurons, conditional knockout ErbB4 from PV neurons promoted vestibular compensation. Further investigation showed that vestibular compensation could be promoted by chemogenetic inhibition of contralateral MVN or activation of ipsilateral MVN PV neurons. Additional neural tracing study revealed that considerable MVN PV neurons were projecting to the opposite side of MVN, and that activating the ipsilateral MVN PV neurons projecting to contralateral MVN can promote vestibular compensation.

**Conclusion:**

Contralateral MVN PV neuron activation after UL is detrimental to vestibular compensation, and rebalancing bilateral MVN PV neuron activity can promote vestibular compensation, via commissural inhibition from the ipsilateral MVN PV neurons. Our findings provide a new understanding of vestibular compensation at the neural circuitry level and a novel potential therapeutic target for vestibular disorders.

## 1. Introduction

Vestibular disorder is a common clinical symptom characterized by postural imbalance, gaze instability, and vertigo, affecting over 20% individuals yearly, strongly limiting daily activities and the quality of life, and leading to falls or other accidents (Casani et al., 2021). However, some balance system disturbances, including unilateral vestibular hypofunction or loss, can partially ameliorate over time in a process known as “vestibular compensation” (Smith and Curthoys, 1989; Hatat et al., 2022). Investigating the mechanism will facilitate the search for new treatment options for treating vestibular disorders and the comprehension of central nervous system (CNS) plasticity during behavioral recovery (Dieringer, 1995).

The medial vestibular nuclei (MVN) are the primary central target of inner ear afferents, essential in maintaining equilibrium, posture, and head position (Dieringer, 1995; Ris and Godaux, 1998; Barmack, 2003). The activity asymmetry between bilateral MVN underlies the postural and oculomotor deficits induced by unilateral labyrinthectomy (UL) (Fisch, 1973), which is assumed due to the imbalance of the reciprocal commissural inhibitory system of bilateral MVN, and the reduction of the afferent input from the lesioned vestibular (Bergquist et al., 2008; Gaal et al., 2015; Magyar et al., 2022). However, no direct evidence supports the efficacy of commissural system rebalancing in vestibular compensation, and the cellular and neural circuitry mechanism is largely unknown (Paterson et al., 2004; Olabi et al., 2009).

GABAergic neurons in MVN can be classified into subtypes according to the expressed markers (Dutia et al., 1992; Hong et al., 2008; Zeeh et al., 2013). Parvalbumin (PV) is a Ca^2+^ binding protein, and PV-positive neurons are a crucial subtype of GABAergic neurons, characterized by their fast-spiking property and their projecting to the soma or axon initial segments to other neurons (Heizmann, 1993; Arif, 2009; Permyakov and Uversky, 2022). ErbB4 is a tyrosine kinase receptor of neuregulin 1, a neurotrophic growth factor, the majority of PV neurons co-localize with ErbB4 in many brain regions (Bean et al., 2014), and ErbB4 is required to maintain PV neurons’ normal function (Lu et al., 2014). It has been reported that PV expression exhibits asymmetric changes during vestibular compensation (Hong et al., 2008); nevertheless, it is unclear whether MVN PV neurons are involved in vestibular compensation. In the present study, we observed the MVN PV neurons project to the other side of MVN, and this projection mediates vestibular compensation via commissural inhibition.

## 2. Materials and methods

### 2.1. Animals

One hundred forty-five male C57BL/6J mice were purchased from Charles River, China. PV-Cre and loxP-flanked ErbB4 (Floxed-ErbB4) mice have been described previously (Wen et al., 2010; Yi et al., 2020) and backcrossed with C57BL/6J for over 10 generations.

PV-Cre mice were crossed with floxed-ErbB4 mice to generate PV-Cre; floxed-ErbB4^+/+^ (ErbB4 KO) mice, in which ErbB4 was ablated in PV neurons (Yi et al., 2020). Forty-eight PV-Cre or ErbB4 KO mice were used for behavior analysis, most of which (~70%) were male.

Less than 5 mice were housed per cage at 22°C–24°C and 55%–80% humidity, on a 12:12 h light/dark cycle, with water and food available. The Animal Care and Use Committee of Huazhong University of Science and Technology approved all experiments.

### 2.2. Unilateral labyrinthectomy

UL has been previously described (Campos-Torres et al., 2005; Chen et al., 2019; Simon et al., 2020; Magyar et al., 2022) in detail. Briefly, after adult male mice (8–10 weeks) were anesthetized with sodium pentobarbital (50 mg/kg, i.p.), an incision was made behind the ear, and the tympanic membrane, malleus, incus, and stapes were surgically removed. A crooked syringe needle tip was inserted into the oval window for mechanical damage and then rinsed with 100% ethanol for chemical demolition of the vestibule. The space created by labyrinthectomy was filled with a hemostatic sponge. For the sham group, the tympanic membrane, malleus, incus, and stapes were removed without damaging the vestibule.

### 2.3. Behavioral analysis

Only the adult male mice (8–10 weeks) were used in behavioral tests. C57BL/6J mice were acclimatized for 7 days after purchase. All mice were handled in the testing room for at least 30 min per day for 3 days before taking behavioral measurements. Tests of static compensation include scoring for posture asymmetry, spontaneous nystagmus, and head tilt. One day prior to UL and one to 5 days following UL, the tests were performed at the same time of day. Blinded assessors scored the static symptoms.

### 2.4. Posture asymmetry

Posture deficits were scored as previously described (Chen et al., 2019): 10 points for spontaneous barrel rolling; 9 points for barrel rolling elicited by a light touch or puff of air; 8 points for the recumbent position on the deafferented side without leg support; 7-6 points for some ipsilateral leg support; 5 points for moving with bilateral leg support; 4-3 points for occasional postural asymmetry; 2-1 points for barely perceptible postural asymmetry.

### 2.5. Spontaneous nystagmus

Electronystagmography was recorded by inserting electrodes into the nasal and lateral orbital margins of the two eyes. The frequency of the fast-phase pullback of the cornea-retinal potential was used to evaluate spontaneous nystagmus (Pietkiewicz et al., 2012).

### 2.6. Head tilt

Head tilt was measured based on the angle between the line from the center of the sacrum to the center of the first thoracic vertebra and the line from the tip of the nose to the center of the parietal.

### 2.7. Western blot

The medial vestibular nuclei were isolated from both sides after the mice were anesthetized. Tissues were homogenized in ice-cold RIPA buffer (Beyotime Biotechnology) supplemented with proteinase inhibitors and subjected to centrifugation at 12,000 rpm for 15 min at 4°C to collect the supernatant. SDS-PAGE separated proteins were transferred to PVDF membranes. After being blocked with tris-buffered saline (TBS) containing 0.1% Tween-20, and 5% nonfat powdered milk for 1 h at room temperature, PVDF membranes were incubated with primary antibodies overnight at 4°C with antibody dilution buffer (Elabscience, Cat. No. E-IR-R125). After washing, the PVDF membrane was incubated with horseradish peroxidase (HRP)-labeled secondary antibodies (AS014, ABclonal, 1:10000) in TBS and 0.1% Tween-20 for 1 h at room temperature. Signal revelation was performed using an enhanced chemiluminescence substrate (RM00021, ABclonal) detection system. Band intensities were scanned using MicroChemi 4.2 (DNR Bio-imaging Systems, Israel), and quantified using ImageJ (National Institutes of Health, United States). Primary antibodies used were: rabbit anti-α-tubulin (AC003, ABclonal, 1:4000), rabbit anti-parvalbumin (A2791, ABclonal, 1:800), and rabbit anti-ErbB4 (A10853, ABclonal, 1:800), and rabbit anti-GABA (A2052, Sigma, 1:50).

### 2.8. Immunofluorescence

Mice were anesthesia with sodium pentobarbital (50 mg/kg, i.p.) and perfused transcardially with 0.1 mol/L phosphate-buffered saline (PBS) and 4% paraformaldehyde (PFA) in PBS. After post-fixed at 4°C overnight with PFA and gradient dehydration with 15% and 30% sucrose solution, brains were frozen in OCT medium (Tissue-Tek, Sakura, Japan), and sliced at 35 μm using a Leica cryostat (Thermo Scientific, HM550). After washing with PBS five times, slices were mounted to a slide with anti-florescence decay sealant containing DAPI (Solarbio, China). Fluorescent signals were imaged with Olympus Fluoview FV1000. Five films were analyzed per mouse, and each group contained 3 mice.

### 2.9. Stereotaxic viral injection

Adult mice were anesthesia with sodium pentobarbital (50 mg/kg, i.p.) and head-fixed in a stereotaxic device (RWD life science; 68025). Viruses were injected into the unilateral medial vestibular nucleus coordinates relative to bregma: anteroposterior, −6.05 mm; mediolateral, ±0.75 mm; dorsoventral, −4.3 mm using a glass pipette (Cetin et al., 2006) (300 nL per mouse, 50 nL/min). After injection, the glass pipette was left in place for 15 min and removed slowly. The skin was sutured and sterilized with iodophors. The titers of AAV-EF1α-DIO-GCaMP6m-WPRE-hGH-polyA (Brain VTA, PT-0283), AAV-EF1α-DIO-hM3Dq-EGFP-WPRE (Obio Technology, Shanghai, H15959), AAV-EF1α-hM4Di-EGFP-WPRE (Obio Technology, Shanghai, H15963), AAV-retro-hSyn-DIO-hM3Dq-EGFP (GeneChem technologies, AAV0071), AAV-retro-CMV-DIO-EGFP (ViGene Biosciences, AV200001) were 10^12^ genome copies per mL. Mice were injected with clozapine-N-oxide [CNO, dissolved in saline 3 mg/kg, i.p.; 10 μM, 1 μL per mouse, guide cannula, (coordinates relative to bregma: anteroposterior, −6.05 mm; mediolateral, ±0.75 mm; dorsoventral, −4.3 mm)] or vehicle to active hM3Dq or hM4Di, guide cannula was implanted 100 μm above the MVN (coordinates relative to bregma: anteroposterior, −6.05 mm; mediolateral, 0.75 mm; dorsoventral, −4.2 mm), 30 min before the behavioral tests.

### 2.10. *In vivo* calcium imaging

The procedures have been described previously (Chen et al., 2021). To record contralateral MVN PV neuron’s activity after UL, PV-Cre mice were injected with viruses (AAV-EF1α-DIO-GCaMP6m-WPRE-hGH-polyA) in contralateral MVN. Three weeks later, a gradient-index (GRIN) lens (0.5 mm in diameter and 5.7 mm in length, Fsphotonics Technology, Shanghai, China) was placed above MVN with 100 μm. The GRIN lens was fixed on the skull with dental cement. After a day of recovery, mice underwent UL and recorded 0 h, 1 h, 3 h, 6 h in contralateral MVN. During recording, the distance of the microscope to the GRIN lens was adjusted by a micro-manipulator until the field of view was in focus. Images were collected at 30 frames per second using the MiniScope V2.0 software.

Images were processed using MATLAB R2020b (Mathworks, Natick, MA) with customized code (https://github.com/thinkertech333/analysisforminiscope, Thinkertech, Nanjing, China), and Cellsort 4.0 software. After motion correction (set to 10) and denoising (set to 100), the region of interest was manually selected according to the fluorescence intensity. The Ca^2+^ signals in the 0 h were considered a baseline and the average Ca^2+^ signal in this period was used as a reference (*F*_0_) to normalize the fluorescence signal (Δ*F*/*F*). The formula is as the following:


$$\Delta F/F\;=\;\frac{F_{\mathrm{signal}}-F_{0}}{F_{0}}$$

*F*_signal_ is the real-time Ca^2+^ signal intensity of the PV neurons of interest, and *F*_0_ is the average Ca^2+^ signal intensity in the baseline period.

### 2.11. Statistical analysis

Data were analyzed by paired or unpaired *t*-tests, one-way ANOVA followed by Tukey’s test, or two-way ANOVA followed by the Bonferroni test, using GraphPad Prism 8.00 (GraphPad Software) software. Data are expressed as the mean ± SEM, and statistical significance was considered when *p* < 0.05.

## 3. Results

### 3.1. The activity of PV neurons is selectively elevated in the contralateral MVN after UL

**Figure 1:**
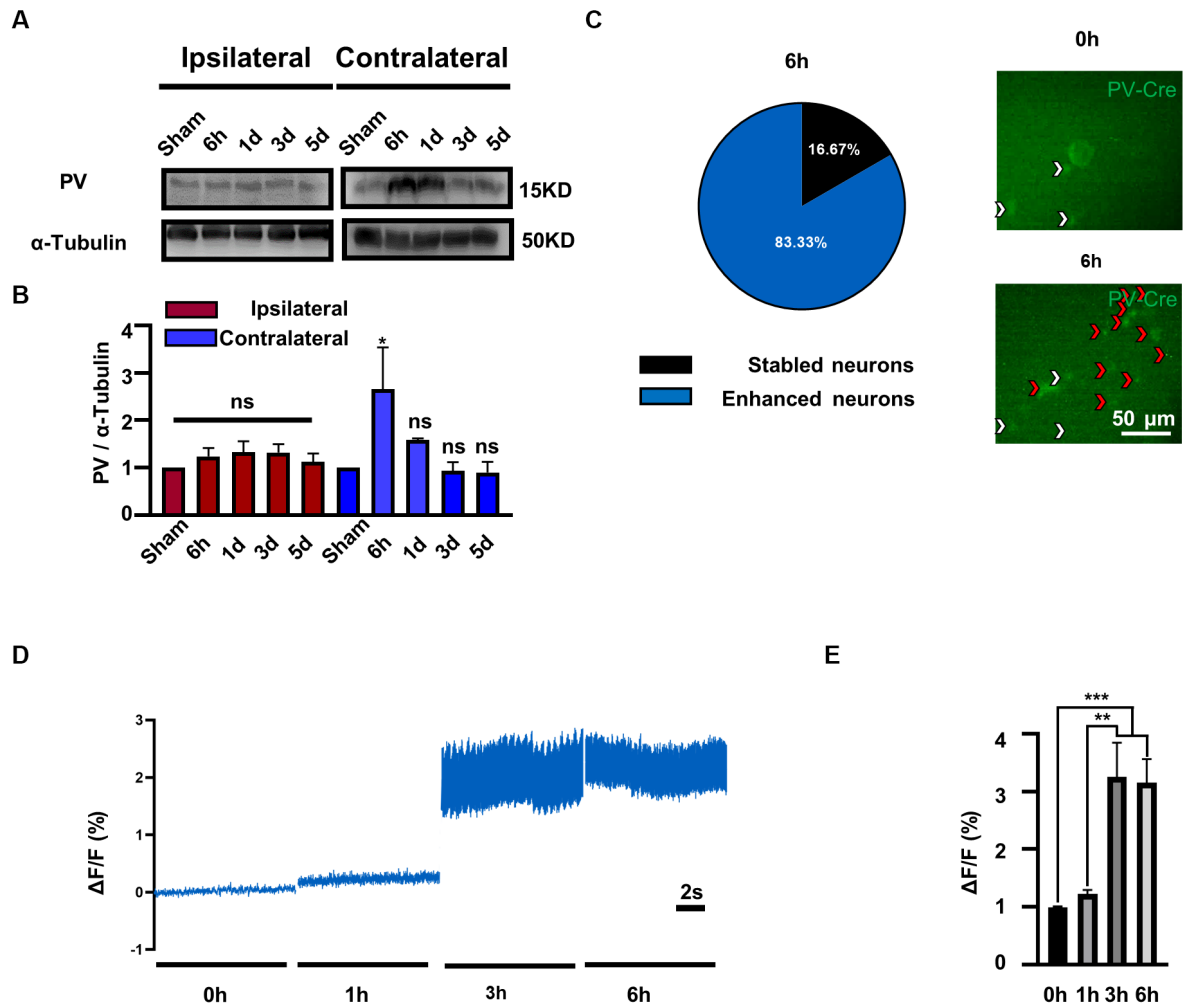
PV expression and PV neurons activity is selectively increased in contralateral MVN after UL. **(A)** Representative western blotting images of PV in the bilateral MVNs after UL. α-tubulin was used as the loading control. **(B)** Quantitative analysis of PV in the bilateral MVNs in **(A)**. *n* = 3 per group. One-way ANOVA, *F* (4, 15) = 3.231, *p* = 0.0423; Dunnett’s *post hoc* test: Sham vs. 6 h, *p* = 0.0427. ^*^p < 0.05, ns, no significant difference. **(C)** Most PV neurons in the contralateral MVN activated 6 h after UL (83.3%). Representative *in vivo* calcium imaging photomicrographs of PV neurons in contralateral MVN 0 h and 6 h shown on the right, white arrows indicate stable neurons and red arrows indicate activity enhanced neurons. **(D,E)** PV neurons activity in the contralateral MVN increased after UL, revealed by *in vivo* calcium imaging. **(D)** Representative traces of Ca^2+^ signals from PV neurons in contralateral MVN after UL 0 h to 6 h. **(E)** Quantification of Δ*F*/*F* in **(D)**. *n* = 20 neurons from 3 mice. One-way ANOVA, *F* (3, 76) = 11.05, *p* < 0.0001; Tukey’s *post hoc* test, 0 h vs. 3 h, *p* = 0.0002; 0 h vs. 6 h, *p* = 0.0004; 1 h vs. 3 h, *p* = 0.0011; 1 h vs. 6 h, *p* = 0.0021. ^**^p < 0.01 and ^***^p < 0.001.

To investigate the role of PV in bilateral MVN after UL, we first evaluated the expression of PV protein by western blotting. Compared to the sham group, PV expression was increased in the contralateral rather than ipsilateral MVN at 6 h after UL, (Figures 1A,B), one-way ANOVA, [*F* (4, 15) = 3.231, *p* = 0.0423], returning to baseline at 1 day after UL.

To further validate whether enhanced protein expression is associated with PV neurons activity, we observed the calcium signals of contralateral MVN PV neurons. Interestingly, we discovered that the majority of PV neurons (83.3%) (Figure 1C) displayed enhanced activity in 3–6 h after UL [Figures 1D,E, One-way ANOVA, *F* (3, 76) = 11.05, *p* < 0.0001]. In comparison, the other (16.7%) maintained a stable activity throughout the procedure (data not shown). Together, these results suggest that the expression of PV protein may be selectively increased in the contralateral MVN and accompanied by increased PV neuron activity.

### 3.2. ErbB4 KO can promote vestibular compensation

**Figure 2:**
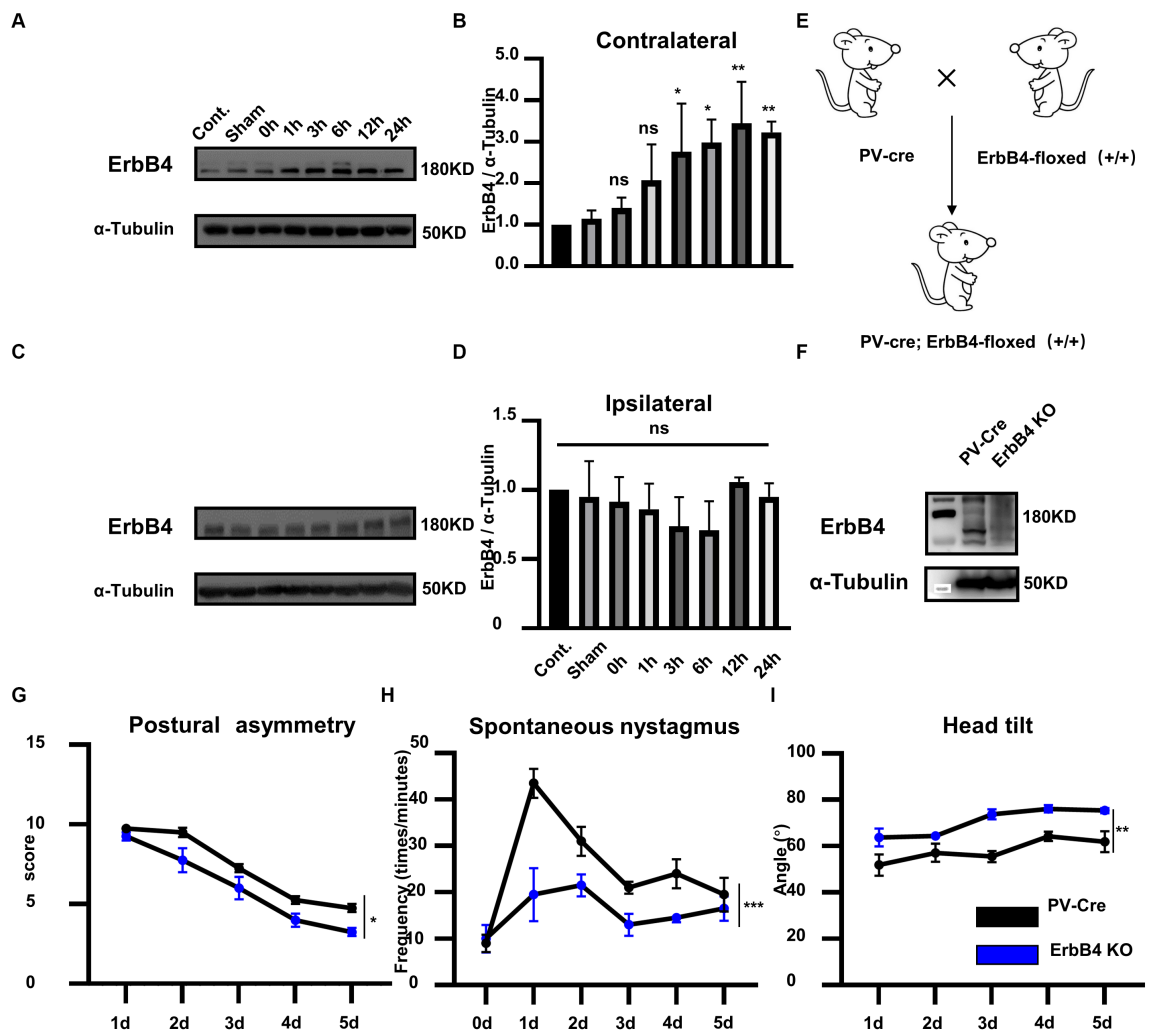
ErbB4 in MVN PV neurons is detrimental to the vestibular compensation. **(A–C)** Western blot for ErbB4 in the bilateral MVNs after UL 0 h, 1 h, 3 h, 6 h,12 h, 24 h, 3 days, and control, sham. α-tubulin served as a loading control. **(B)** Quantitative analysis of the expression of ErbB4 in contralateral MVN in **(A)**. Data are expressed as mean ± SEM, and there are 3 to 5 mice in each group. One-way ANOVA, *F* (7, 21) = 8.604, *p* < 0.0001; Tukey’s *post hoc* test: cont. vs. 1 h, *p* = 0.487; cont. vs. 3 h, *p* = 0.0486; cont. vs. 6 h, *p* = 0.0115; cont. vs. 12 h, *p* = 0.0013; cont. vs. 24 h, *p* = 0.007. One-way ANOVA, ^*^p < 0.05 and ^**^p < 0.01, ns, no significant difference. **(D)** Quantitative analysis of the expression of ErbB4 in ipsilateral MVN in **(C)**. Data are expressed as mean ± SEM, and there are three mice in each group. One-way ANOVA, Bonferroni’s *post hoc* test, ns, no significant. **(E)** Schematic illustrator of PV-Cre; ErbB4-floxed^+/+^ (ErbB4 KO). **(F)** Western blot for ErbB4 in the MVN in ErbB4 KO compared with PV-Cre mice. α-tubulin served as a loading control. **(G–I)** The behavior detection after UL in ErbB4 KO mice. **(G)** Postural asymmetry, *F* (1, 6) = 6.919, *p* = 0.039; **(H)** spontaneous nystagmus, *F* (1, 6) = 39.94, *p* = 0.0007; **(I)** head tilt, *F* (1, 6) = 21.2, *p* = 0.0037. *n* = 4 mice in PV-Cre and WT group, respectively. Two-way ANOVA, Bonferroni’s *post hoc* test. ^*^p < 0.05, ^**^p < 0.01, and ^***^p < 0.001.

To investigate whether ErbB4 is involved in vestibular compensation, we first detected the alterations in ErbB4 protein expression in MVN following UL. Compared with the control group, the expression of ErbB4 protein significantly increased in the contralateral MVN at 3–24 h after UL, while there was no significant change in the ipsilateral MVN [Figures 2A–D, one-way ANOVA, *F* (7, 21) = 8.604, *p* < 0.0001 for contralateral]. We next examined the mice that specifically knock out ErbB4 in PV neurons (PV-Cre; ErbB4-floxed^+/+^, ErbB4 KO) (Figures 2E,F), thereby attenuating the activation of PV neurons in both sides of MVN after UL. ErbB4 KO mice exhibited faster vestibular compensation, [Figures 2G–I, postural asymmetry, *F* (1, 6) = 6.919, *p* = 0.039; spontaneous nystagmus, *F* (1, 6) = 39.94, *p* = 0.0007; head tilt, *F* (1, 6) = 21.2, *p* = 0.0037], suggesting ErbB4 in MVN PV neurons is detrimental to the vestibular compensation.

### 3.3. Inhibition of PV neurons activity in contralateral MVN can promote vestibular compensation

**Figure 3:**
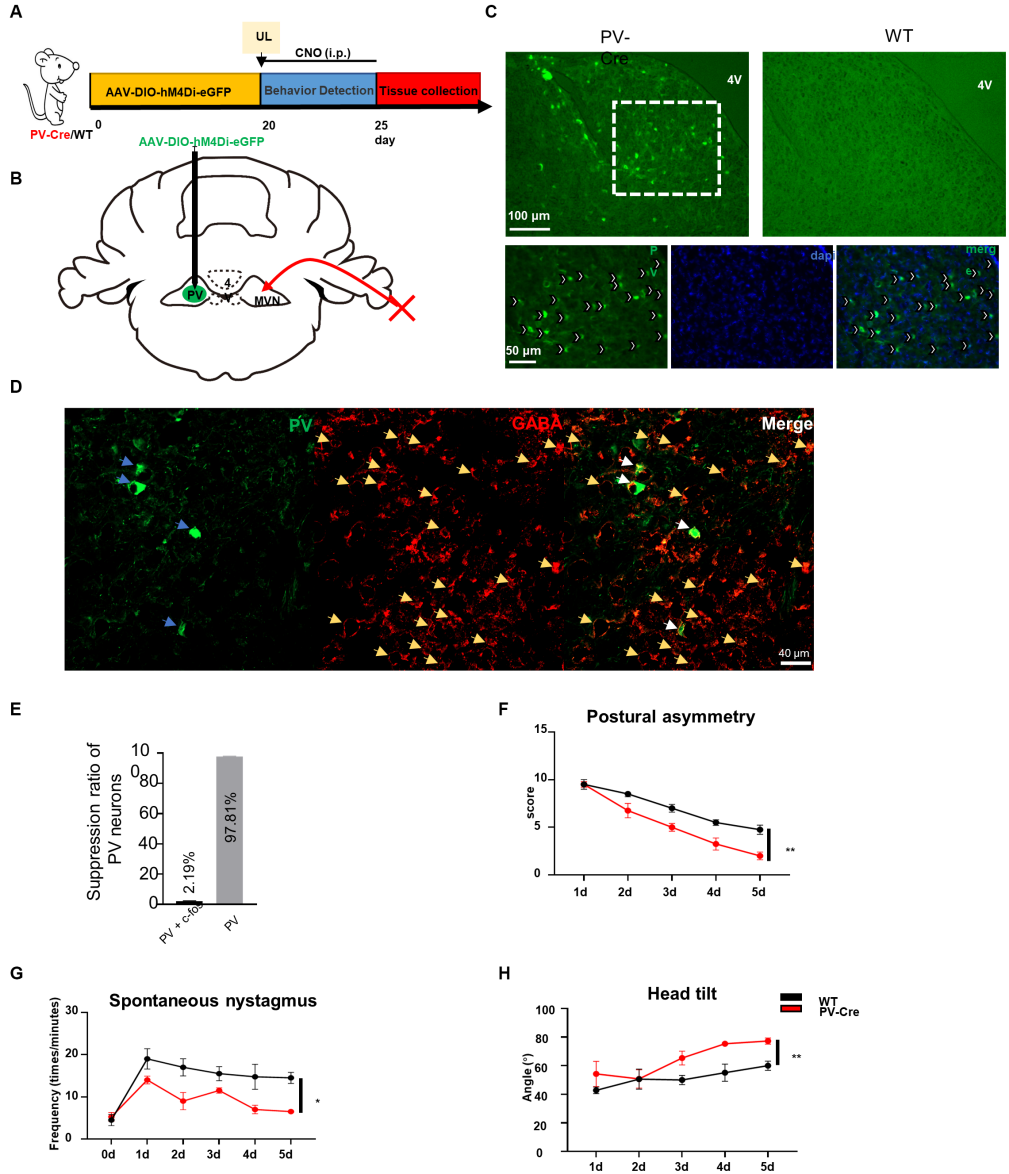
Inhibition of contralateral MVN PV neurons promotes vestibular compensation. **(A)** Schematic experimental design. Twenty days after AAV-hSyn-DIO-hM4Di-eGFP stereotaxic injection into contralateral MVN of PV-Cre mice, UL was conducted followed by CNO i.p. injection twice every day. Vestibular dysfunction was evaluated within 1–2 h after injecting of CNO every day. **(B)** Schematic illustration of AAV injection in contralateral MVN of PV-Cre mice. **(C)** Representative photomicrographs showing PV neurons in contralateral MVN after injecting the virus. Images at the bottom are magnified images of the boxed regions in the upper image. Arrows (in white) indicate PV neurons. Scale bar, top, 100 μm; bottom, 50 μm. **(D)** Representative image of co-staining of PV and GABAergic neurons. **(E)** The percentage of PV neuron inhibition after CNO injection. **(F–H)** Inhibiting the contralateral MVN PV neurons promotes vestibular compensation. **(F)** Postural asymmetry, *F* (1, 6) = 13.97, *p* = 0.0096; **(G)** spontaneous nystagmus, *F* (1, 6) = 11.18, *p* = 0.0156; **(H)** head tilt, *F* (1, 6) = 20.93, *p* = 0.0038. *n* = 4 mice in PV-Cre and WT group, respectively. Two-way ANOVA, Bonferroni’s *post hoc* test. ^*^p < 0.05 and ^**^p < 0.01.

To confirm whether the hyperactivation of PV neurons in contralateral MVN is detrimental to the vestibular compensation, we employed a chemogenetic technique to specifically inhibit the activity of PV neurons in the contralateral MVN after UL. PV-Cre mice were injected with AAV-EF1α-hM4Di-EGFP-WPRE virus in the contralateral MVN (Figures 3A–E), and wild-type mice were used as control. Twenty days later, these mice were subjected to UL followed by CNO i.p. injection for five days (once the mice had undergone UL, CNO was given immediately). Behavior tests were performed 30 min after CNO administration. For the control mice, vestibular symptoms were gradually alleviated, including postural asymmetry, spontaneous nystagmus, and head tilt; interestingly, in PV-Cre mice, all these behavior symptoms were alleviated faster than wild-type mice [Figures 3F–H, postural asymmetry, *F* (1, 6) = 13.97, *p* = 0.0096; spontaneous nystagmus, *F* (1, 6) = 11.18, *p* = 0.0156; head tilt, *F* (1, 6) = 20.93, *p* = 0.0038], suggesting inhibition of contralateral MVN PV neurons effectively accelerated vestibular compensation.

### 3.4. Activation of PV neurons activity in ipsilateral MVN can promote vestibular compensation

**Figure 4:**
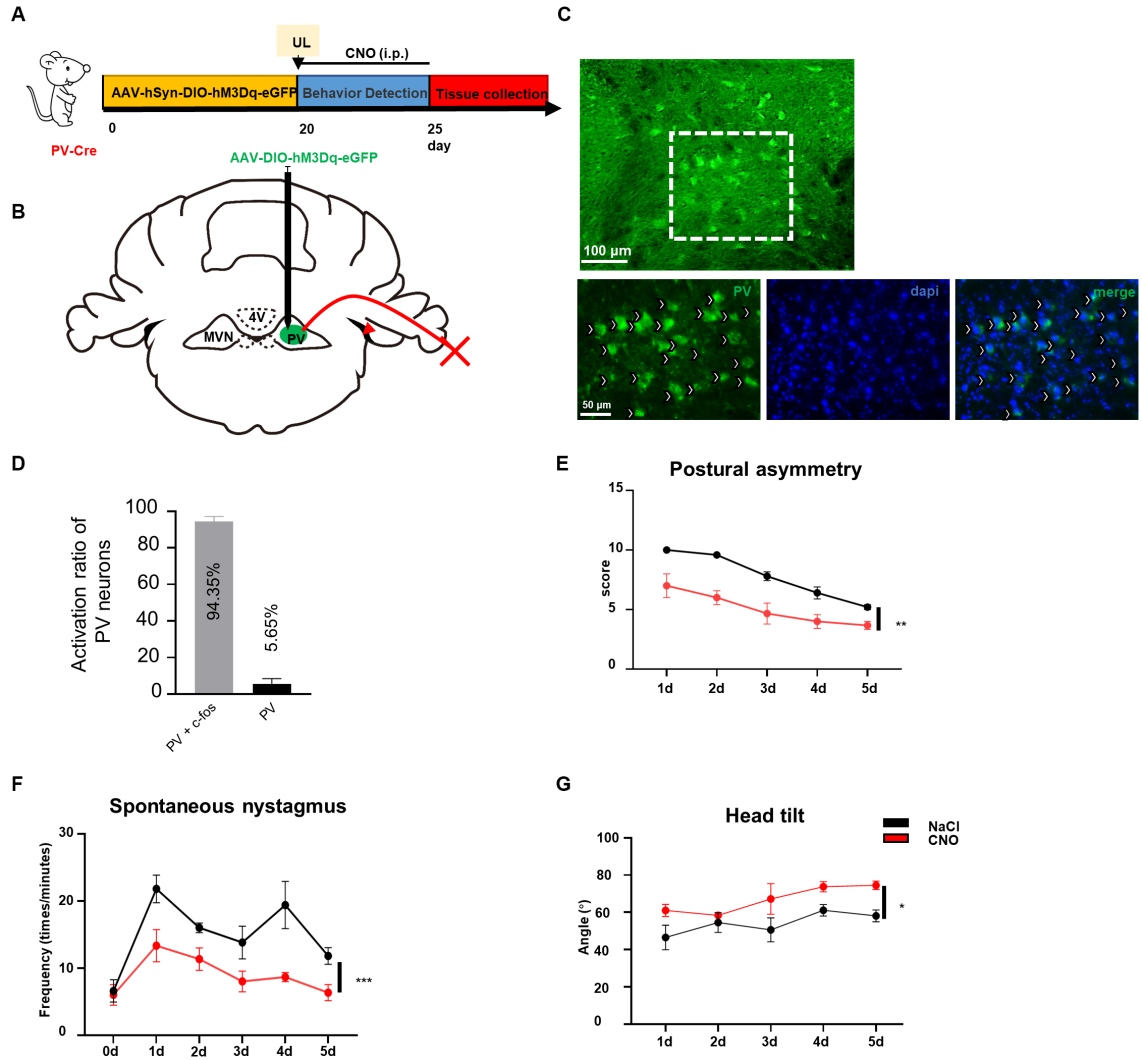
Activation of ipsilateral vestibular PV neurons can improve vestibular compensation. **(A)** Schematic experimental design for PV-Cre. **(B)** Schematic illustration of AAV-hSyn-DIO-hM3Dq-eGFP injection in ipsilateral MVN of PV-Cre mice. **(C)** Representative photomicrographs showing PV neurons in ipsilateral MVN after injecting the virus. Arrows (in white) indicate PV neurons. Scale bar, top, 100 μm; bottom, 50 μm. **(D)** The percentage of PV neuron activation after CNO injection. **(E–G)** The behavior detection after UL by activating the ipsilateral MVN PV neurons. **(D)** Postural asymmetry, *F* (1, 6) = 26.05, *p* = 0.0022; **(E)** spontaneous nystagmus, *F* (1, 6) = 35.75, *p* = 0.001; **(F)** head tilt, *F* (1, 6) = 11.08, *p* = 0.0158. *n* = 4 mice in PV-Cre and WT group, respectively. Two-way ANOVA, Bonferroni’s *post hoc* test. ^*^p < 0.05, ^**^p < 0.01, and ^***^p < 0.001.

Previously we have demonstrated that reducing the activity of PV neurons in the contralateral MVN is beneficial to vestibular compensation, it needs to be further investigated whether vestibular compensation could also be promoted by enhancing the activity of ipsilateral MVN PV neurons to achieve a new balance between bilateral MVN PV neurons. By injecting AAV-EF1α-DIO-hM3Dq-EGFP-WPRE virus into the ipsilateral MVN of PV-Cre mice, and intraperitoneal injecting CNO (Figures 4A–D), we found that for the wild-type control mice, spontaneous vestibular compensation could be observed 1–5 days after UL, however, activation of PV neurons in the ipsilateral MVN could also further promote vestibular compensation [Figures 4E,G, postural asymmetry, *F* (1, 6) = 26.05, *p* = 0.0022; spontaneous nystagmus, *F* (1, 6) = 35.75, *p* = 0.001; head tilt, *F* (1, 6) = 11.08, *p* = 0.0158]. Together, these results suggest that a new balance of activity in bilateral MVN PV neurons contributes to the amelioration of static deficits after UL.

### 3.5. PV neurons project to the contralateral MVN

**Figure 5:**
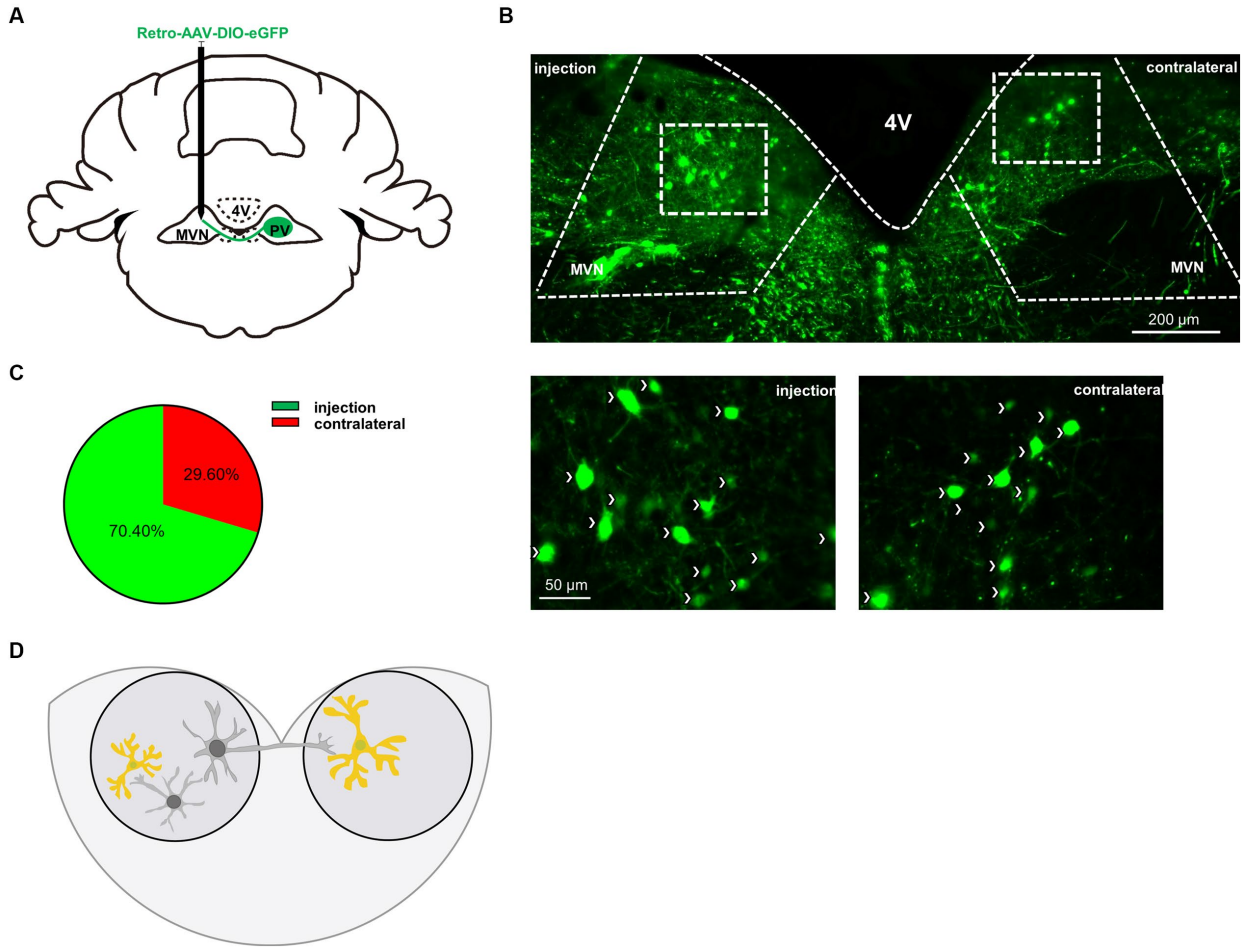
PV neurons project to the contralateral MVN. **(A)** Schematic illustration of AAV-retro-CMV-DIO-EGFP injection in unilateral MVN of PV-Cre mice to trace the soma in contralateral MVN. **(B)** Representative photomicrographs showing EGFP-expression PV neurons in bilateral MVNs after virus injection. Arrows (in white) indicate PV neurons. Scale bar, top, 200 μm; bottom, 50 μm. **(C)** Quantification the distribution of bilateral MVNs PV neurons, *n* = 3 mice. **(D)** Schematic illustration of PV neurons in MVN.

Vestibular commissures connections are essential in vestibular compensation and are mediated predominantly by inhibitory GABAergic neurons (Gliddon et al., 2005; Malinvaud et al., 2010). To verify whether PV neurons project from one side of MVN to the other, we injected AAV-retro-CMV-DIO-EGFP virus into one side of MVN (Figure 5A). Twenty-eight days later, we found PV neuron projections from the other side of MVN (Figure 5B). Next, we counted the number of PV neurons in bilateral MVN, the injection side accounted for 70.4% of the total, while the contralateral accounted for 29.6% (Figure 5C), suggesting considerable PV neurons in MVN projecting to the opposite side MVN, though we could not exclude the possibility of PV neurons may also innervate other neurons in the same side of MVN. These results suggest PV neurons may influence vestibular compensation through commissural inhibition (Figure 5D).

### 3.6. Activation of PV neurons from ipsilateral to contralateral MVN promotes vestibular compensation

**Figure 6:**
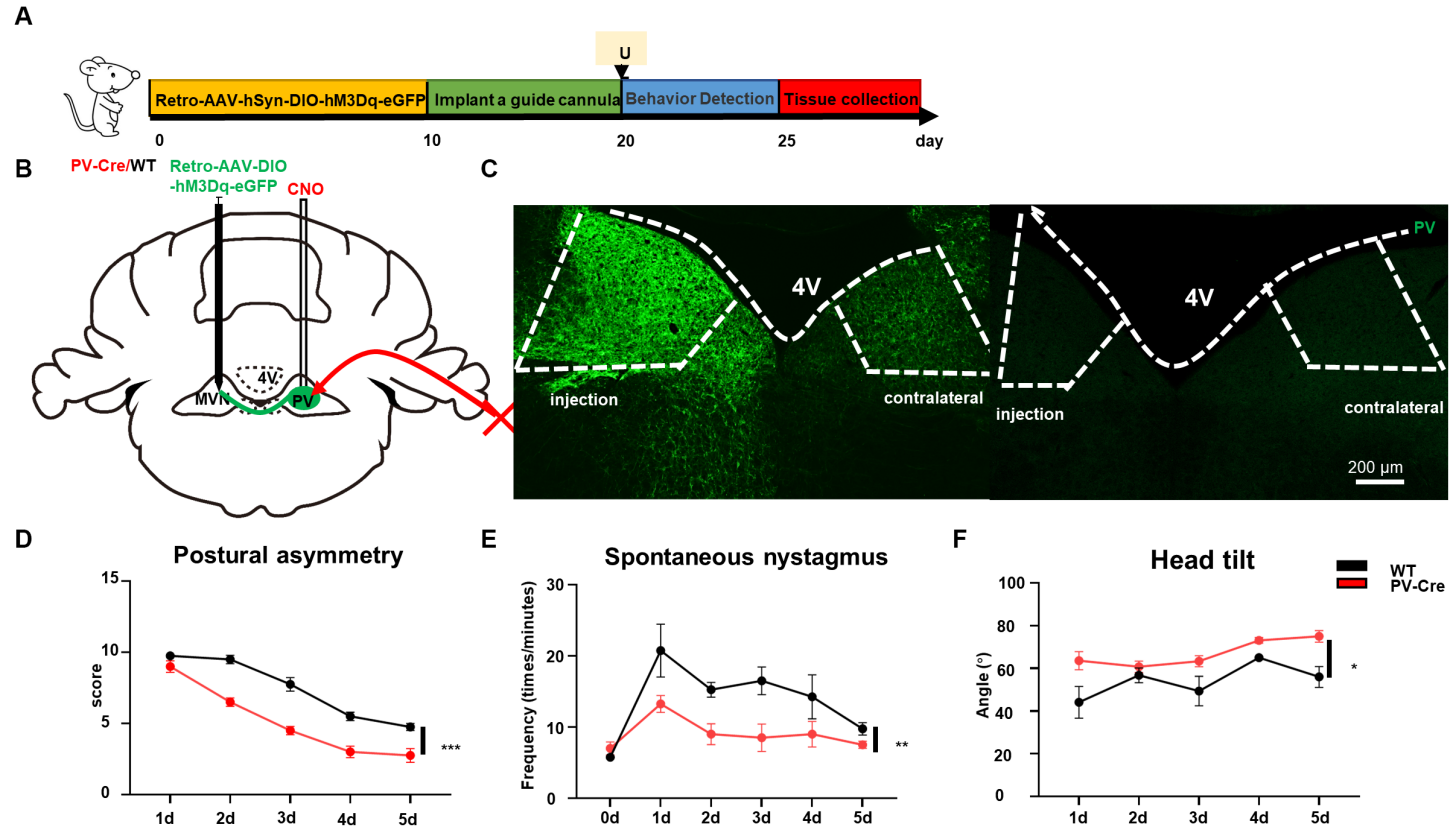
Activation of commissural PV neurons can change vestibular compensation. **(A)** Schematic experimental design for PV-Cre and littermates. Illustration of retro-AAV-hSyn-DIO-hM3Dq-eGFP injection in contralateral MVN of PV-Cre mice. **(B)** Schematic illustration of retro-AAV-hSyn-DIO-hM3Dq-eGFP injection in contralateral MVN of PV-Cre mice. **(C)** Representative photomicrographs showing PV-positive neurons in bilateral MVNs after injecting the virus. Scale bar, 200 μm. Schematic illustration of retro-AAV-hSyn-DIO-hM3Dq-eGFP injection in contralateral MVN of PV-Cre mice. **(D–F)** The behavior detection after UL by activating the commissural PV neurons. **(D)** Postural asymmetry, *F* (1, 6) = 53.8, *p* = 0.0003; **(E)** spontaneous nystagmus, *F* (1, 6) = 17.5, *p* = 0.0058; **(F)** head tilt, *F* (1, 6) = 10.7, *p* = 0.017. *n* = 4 mice in PV-Cre and WT group, respectively. Two-way ANOVA, Bonferroni’s *post hoc* test. ^*^p < 0.05, ^**^p < 0.01, and ^***^p < 0.001.

**Figure 7:**
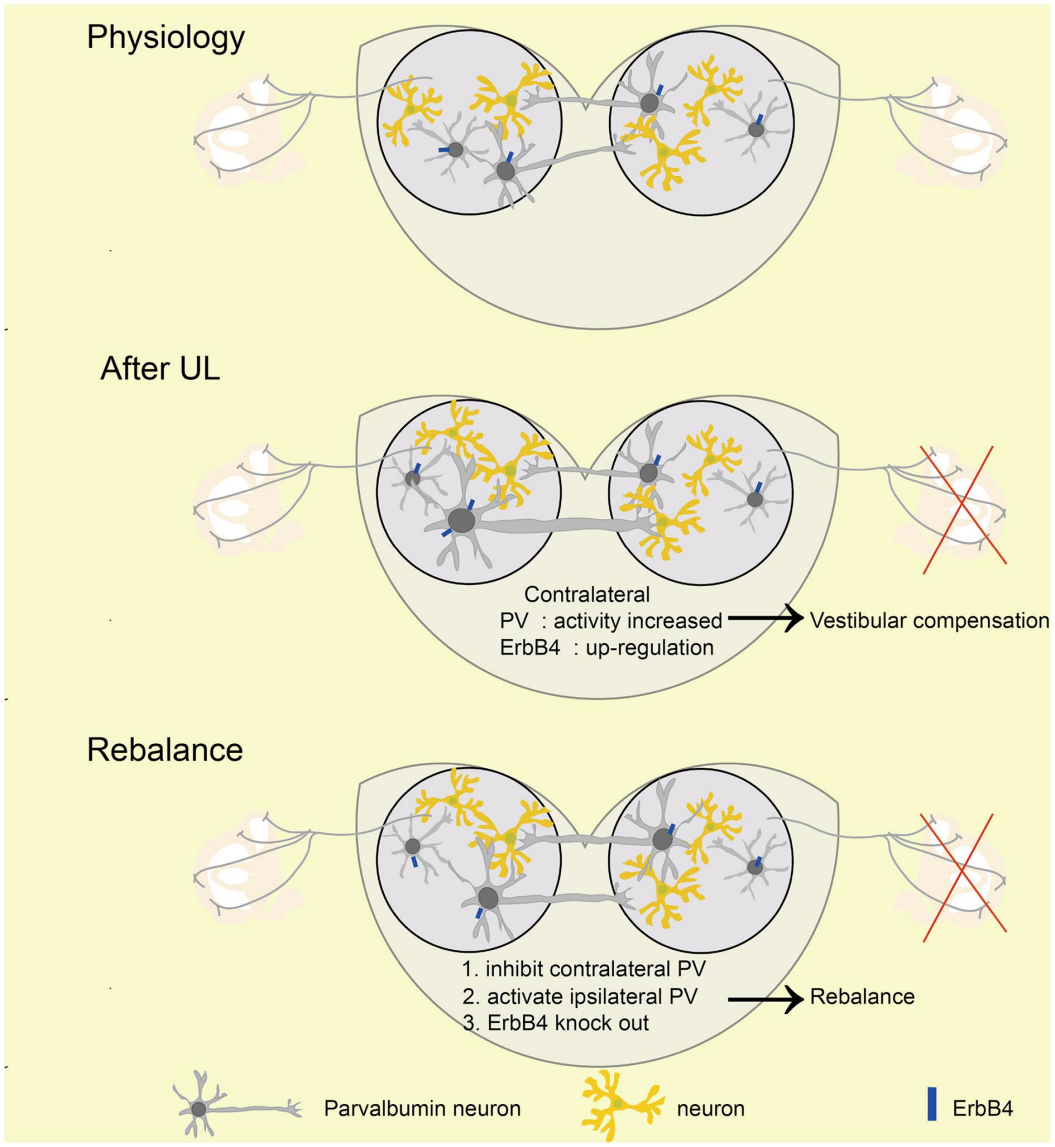
Mechanism of rebalancing MVN PV neurons’ activity promotes vestibular compensation via commissural inhibition Schematic diagrams showing the role of PV neurons in vestibular compensation. In the physiology state, the activity of bilateral MVN neurons is balanced. After UL, loss of afferent from peripheral leads to neuronal silencing in the ipsilateral MVN, which is major caused by sustained and enhanced input from contralateral MVN inhibitory neurons. Activation of ipsilateral or inhibition of contralateral PV neurons, as well as knockout of ErbB4 of PV, could promote the rebalance of the activity of bilateral MVN neurons.

The vestibular commissural inhibitory system is essential for vestibular compensation (Bergquist et al., 2008; Malinvaud et al., 2010). Modulation of the vestibular commissures inhibitory system facilitates vestibular compensation by enhancing ipsilateral to contralateral GABAergic neuron projections (Chen et al., 2019); however, the cell type of these neurons are unknown. We wondered whether ipsilateral MVN PV neurons could contribute to vestibular compensation through this way. The AAV-retro-hSyn-DIO-hM3Dq-EGFP virus was injected into the contralateral MVN, then a guide cannula was implanted into the ipsilateral MVN on day 10. This allowed for the local injection of CNO into one side of MVN and selective activation of only PV neurons that were projecting from ipsilateral to contralateral MVN (Figures 6A–C). Behavioral analysis revealed that activation of these PV neurons promoted vestibular compensation [Figures 6D–F, postural asymmetry, *F* (1, 6) = 53.8, *p* = 0.0003; spontaneous nystagmus, *F* (1, 6) = 17.5, *p* = 0.0058; head tilt, *F* (1, 6) = 10.7, *p* = 0.017]. These results indicate that PV neurons in the ipsilateral MVN regulate the activity of neurons in the contralateral MVN through commissural inhibition between both sides of MVN, and activation of these PV neurons can facilitate vestibular compensation, which may be brought about by the restoration of activity balance in both sides of MVN (Figure 7).

## 4. Discussion

Here, we found that MVN PV-positive GABAergic neurons contribute to vestibular compensation via commissural inhibition. First, we observed that the expression of PV in contralateral but not ipsilateral MVN was up-regulated, and contralateral MVN PV neurons displayed higher activity at 6 h after UL. Second, compromising PV neurons’ function by conditional knockout ErbB4 in PV neurons facilitates vestibular compensation, suggesting contralateral MVN PV neurons’ detrimental role in vestibular compensation. Third, promoting vestibular compensation by chemogenetic inhibition of contralateral or activation of ipsilateral MVN PV neurons raises the importance of activity rebalancing in bilateral MVN PV neurons. Fourth, we found that PV neurons project to the other MVN by circuit tracing, and activation of ipsilateral MVN PV neurons projecting to the other side MVN could also promote vestibular compensation, suggesting PV neurons may be involved in vestibular compensation through the commissural inhibitory system.

PV is widely distributed in the peripheral and central nervous system (MacManus et al., 1982; Heizmann, 1984; Hontanilla et al., 1998; Permyakov and Uversky, 2022), acting as a calcium buffer in the cytosol (Heizmann, 1993). PV expression positively correlates with PV neuron activities, suggesting the importance of PV in buffering the cytosol calcium (Heizmann, 1984; Vreugdenhil et al., 2003). Due to their fast spiking property and their targeting to the soma and axon initial segments of glutamate neurons, PV neurons are the main cell type controlling the local neuron population firing rates, through their GABA release (Schwaller, 2010; Olinger et al., 2012). Consistent with the literature, we observed asymmetric PV expression after UL, and the firing frequency of contralateral PV neurons was increased, suggesting the involvement of PV neurons in vestibular compensation (Hong et al., 2008). It is important to note that the activity of contralateral MVN PV neurons was increased 1 h after UL, observed by *in vivo* calcium imaging. Further investigation of the pathophysiological procedure during the first hour after UL will be crucial for understanding postlesion plasticity in the adult CNS (Lacour et al., 2016). *In vivo* calcium imaging results also show that the activity of contralateral MVN PV neurons is greatly increased during 3 h and 6 h after UL, consistent with the PV overexpression, suggesting the importance of contralateral PV neurons during the first several hours. Though the PV expression of contralateral MVN resumed to normal 1 day after UL, we cannot exclude the possibility that the contralateral MVN PV neurons are still involved in the postlesion plasticity.

For the *in vivo* calcium imaging experiment, we did not observe the size difference between the active and dormant PV neurons, due to technological limitations that we cannot label the dormant PV neurons *in vivo*. According to the literature, it has been reported that the long-range projecting PV neurons exhibit morphology and intrinsic electrophysiological properties similar to local PV neurons (Lee et al., 2014; Bertero et al., 2020); however, in the auditory cortex, long-range projecting PV neurons exhibit a higher expression of a subtype of voltage-sensitive potassium channel (i.e., Kv1.1) than local PV neurons and thus were less excitable (Zurita et al., 2018). We speculate that the dormant PV neurons in the calcium imaging experiment might be more long-range projecting PV neurons, and on the contrary, the active PV neurons might be more local. However, in the last experiment (Figure 6), we specifically activate the long-projecting PV+ neurons from ipsilateral to contralateral MVN, which promotes the vestibular compensation, suggesting the possibility that at least some of the activated PV neurons in the calcium imaging experiment should be long-range projecting PV neurons.

To further investigate PV neurons’ hyperactivation in contralateral MVN, ErbB4 was specifically knocked out from PV neurons. ErbB4 is the receptor of neurotrophic factor neuregulin 1, which is required to maintain PV GABA release (Sweeney et al., 2000; Buonanno, 2010; Lu et al., 2014). Interestingly, vestibular compensation is promoted, compared to PV-Cre mice as control. More importantly, we observed the expression of ErbB4 was increased 3 h after UL, and maintained overexpression for more than 24 h. Considering that ErbB4 is expressed in GABAergic neurons, and mainly in the PV-positive subtype (Fazzari et al., 2010; Bernard et al., 2022), ErbB4 in MVN PV neurons might play a certain detrimental role in vestibular compensation more than 1 day.

To further confirm the causal relationship of contralateral MVN PV neurons in compromising vestibular compensation, these neurons were chemogenetically inhibited after UL once a day for 5 days by CNO administration. The first CNO administration was given immediately after UL, however, the behavior phenotypes induced by UL were not alleviated on day one, suggesting contralateral MVN PV neurons might be involved in vestibular compensation 1 day after UL, which was consistent with ErbB4 overexpression in MVN. Because PV neurons are critical for neural population activity (Hu et al., 2014; Contractor et al., 2021), and the behavior phenotypes induced by UL are related to the unbalance of bilateral MVN activities (Olabi et al., 2009), we expected improving vestibular compensation by activating the ipsilateral MVN PV neurons. Once a day chemogenetic activation of ipsilateral MVN PV neurons after UL for 5 days can also improve vestibular compensation, suggesting the balance of MVN PV neurons activity is crucial for maintaining the vestibular normal function.

PV neurons in the cortex and hippocampus are mainly locally projected GABAergic neurons (Szabo et al., 2022; Zhang et al., 2022). However, long-range GABAergic projections exist from PV neurons in the cortex to subcortical regions of the brain. In the hippocampus, CA3 and CA1 receive PV neuron projections from the entorhinal cortex (Melzer et al., 2012; Basu et al., 2016; Melzer et al., 2017) and medial septum (Freund and Antal, 1988); and in the amygdala, many of the intercalated cells are PV, projecting to the perirhinal, entorhinal, and piriform cortex (Bienvenu et al., 2015). PV neurons in sensory-motor cortical regions project to the striatum, including the somatosensory cortex (Jinno and Kosaka, 2004), auditory cortex (Rock et al., 2016; Bertero et al., 2020), and primary motor cortex (M1) (Melzer et al., 2017). PV neurons in the associative cortex, and the medial prefrontal cortex also project to the striatum, especially the nucleus accumbens (Lee et al., 2014). PV neurons are also involved in cortico-cortical long-range GABAergic projections. Interestingly, according to the present literature, all these PV neurons’ cortico-cortical long-range projections target their contralateral counterparts, including PV neurons in the auditory cortex, visual cortex, and motor cortex (Rock et al., 2018; Zurita et al., 2018). Except for PV neurons, only vasoactive intestinal peptide (VIP)-positive GABAergic neurons in the cortex project to the other side of the counterpart (Bertero et al., 2021) whether PV neurons in MVN can project to the opposite side MVN needs investigation. Retro-AAV virus can infect neurons through the terminal and cell body, and only PV-positive neurons can express eGFP due to the Double-Floxed Inverted Open reading frame in the virus (Tervo et al., 2016). Interestingly, besides the virus injection side, a certain number of eGFP-positive neurons can also be observed on the opposite side of MVN, suggesting MVN PV neurons are an important component in the commissural inhibitory system of MVN. Further investigation illustrated that specifically activation of the commissural MVN PV neurons from the ipsilateral to the contralateral side could also promote vestibular compensation, suggesting besides the local projection, MVN PV neurons are also involved in vestibular compensation through commissural inhibition (Olabi et al., 2009).

MVN has been divided into parvocellular and magnocellular subdivisions (Sekirnjak and du Lac, 2006; Bagnall et al., 2007; Kodama et al., 2012). Here in Figure 3, we observed PV neurons were almost exclusively in the parvocellular division, and importantly, in Figure 5, the MVN PV neurons projecting to the contralateral MVN were also mainly in the parvocellular division, which is consistent with previous findings that GABAergic neurons are mainly located in the parvocellular division (Buttner-Ennever, 1992), and these GABAergic neurons mediate commissural projection (Delfini et al., 2000).

## 5. Conclusion

In conclusion, this study revealed that the balance of MVN PV neurons’ activity via commissural inhibition is essential for vestibular compensation, offering a unique potential therapeutic target for vestibular disorders as well as a new understanding of vestibular compensation at the neuronal circuitry level.

## Data availability statement

The original contributions presented in the study are included in the article/supplementary material, further inquiries can be directed to the corresponding authors.

## Ethics statement

The animal study was approved by Committee of Huazhong University of Science and Technology. The study was conducted in accordance with the local legislation and institutional requirements.

## Author contributions

YZ: Conceptualization, Data curation, Formal analysis, Investigation, Methodology, Software, Visualization, Writing – original draft, Writing – review & editing. GC: Conceptualization, Formal analysis, Software, Writing – review & editing. YaL: Funding acquisition, Writing – review & editing. XL: Writing – review & editing, Formal analysis, Software. HZ: Formal analysis, Software, Writing – review & editing. YiL: Conceptualization, Funding acquisition, Project administration, Resources, Supervision, Validation, Writing – review & editing. BL: Conceptualization, Funding acquisition, Project administration, Resources, Supervision, Validation, Writing – review & editing.
